# m^6^A Modification Mediates Mucosal Immune Microenvironment and Therapeutic Response in Inflammatory Bowel Disease

**DOI:** 10.3389/fcell.2021.692160

**Published:** 2021-08-06

**Authors:** Yongyu Chen, Jing Lei, Song He

**Affiliations:** Department of Gastroenterology, The Second Affiliated Hospital of Chongqing Medical University, Chongqing, China

**Keywords:** m^6^A modification, IBD, immune infiltration, immunotherapy, anti-TNF response

## Abstract

Accumulating evidence links m^6^A modification with immune infiltration. However, the correlation and mechanism by which m^6^A modification promotes intestinal immune infiltration in inflammatory bowel disease (IBD) is unknown. Here, genomic information from IBD tissues was integrated to evaluate disease-related m^6^A modification, and the correlation between the m^6^A modification pattern and the immune microenvironment in the intestinal mucosa was explored. Next, we identified hub genes from the key modules of the m^6^Acluster and analyzed the correlation among the hub genes, immune infiltration, and therapy. We found that IGF2BP1 and IGF2BP2 expression was decreased in Crohn’s disease (CD) tissues and that IGF2BP2 was decreased in ulcerative colitis (UC) tissues compared with normal tissues (*P* < 0.05). m^6^Acluster2, containing higher expressions of IL15, IL16, and IL18, was enriched in M0 macrophage, M1 macrophage, native B cells, memory B cells, and m^6^Acluster1 with high expression of IL8 and was enriched in resting dendritic and plasma cells (*P* < 0.05). Furthermore, we reveal that expression of m^6^A phenotype-related hub genes (i.e., NUP37, SNRPG, H2AFZ) was increased with a high abundance of M1 macrophages, M0 macrophages, and naive B cells in IBD (*P* < 0.01). Immune checkpoint expression in the genecluster1 with higher expression of hub genes was increased. The anti-TNF therapeutic response of patients in genecluster1 was more significant, and the therapeutic effect of CD was better than that of UC. These findings indicate that m^6^A modification may affect immune infiltration and therapeutic response in IBD. Assessing the expression of m^6^A phenotype-related hub genes might guide the choice of IBD drugs and improve the prediction of therapeutic response to anti-TNF therapy.

## Introduction

Inflammatory bowel disease (IBD), including Crohn’s disease (CD) and ulcerative colitis (UC), is a chronic intestinal inflammatory disease (Torres et al., 2017; Ungaro et al., 2017). The precise etiology of IBD remains unclear, but genetic predisposition, disruption of mucosal immune homeostasis, microbial dysbiosis, and environmental factors are involved (Halfvarson et al., 2017; Neurath, 2017). In particular, the immune system is implicated as a major contributor to IBD progression, predominantly through an imbalance between the anti-inflammatory responses of regulatory T cells and M2 macrophages versus the pro-inflammatory responses of M1 macrophages, T helper (Th) 1, Th17, and neutrophils (Canavan et al., 2016; de Souza and Fiocchi, 2016; Zhou et al., 2018, 2019). Current treatments are aimed at relieving inflammation through the use of steroids, biological agents (primarily anti-TNF), and immunosuppressive drugs (Torres et al., 2017; Ungaro et al., 2017). However, approximately 30% of patients using anti-TNF agents do not respond to treatment (Belarif et al., 2019). Thus, the potential mechanism of the progression of IBD needs to be elucidated.

N6-methyladenosine (m^6^A) modification, as the most prevalent RNA epigenetic modification, occurs transcriptionally (Lan et al., 2019; Huang H. et al., 2020). m^6^A methylation is catalyzed by the MTase complex referred to as “writers,” including methyltransferase-like 3/14/16 (METTL3/14/16), RNA binding motif protein 15 (RBM15), KIAA1429, and Wilms tumor 1-associated protein (WTAP; Chen X. Y. et al., 2019), whereas its demethylation is mediated by “eraser” proteins (ALKBH5 and FTO) (Huang Y. et al., 2019; Jin et al., 2020). The m^6^A modification is interpreted by m^6^A “reader” proteins, including the insulin-like growth factor 2 mRNA-binding protein 1/2/3 (IGF2BP1/2/3), YTH-domain family 1/2/3 (YTHDF1/2/3), and HNRNPA2B1 (Huang H. et al., 2018; Jiang et al., 2021). Accumulating evidence suggests that m^6^A modification plays an important role in inflammation, various tumors, innate immunity, and immunotherapy through different m^6^A regulator–related modifications (Chen X. Y. et al., 2019; Wang J. et al., 2019). Most studies focus on various tumors, single regulator, or single immunity cell types, but the influence of m^6^A modification on disease progression has not been examined in IBD.

Recent studies find that m^6^A modification is associated with disease-related immune infiltration. For example, METTL3-mediated m^6^A methylation promotes the activation of dendritic cells (DCs) (Wang H. et al., 2019), and METTL3 leads to an imbalance between Treg cells and native T cells with Treg cells losing the ability to suppress immune and native T cells losing the ability to induce inflammation (Geula et al., 2015; Li et al., 2017). YTHDF1 binds to lysosomal proteases, increases their translational efficiency in DCs, and enhances the tumor infiltrating CD8 + T cell antitumor response (Han et al., 2019). A recent study finds that METTL14 deficiency in T cells promotes spontaneous colitis in mice (Lu et al., 2020). However, the mechanism connecting IBD-related m^6^A modification and immune cell infiltration has not yet been elucidated. Thus, a comprehensive understanding of the different immune infiltrating characteristics induced by m^6^A regulators will improve our understanding of immune regulation in IBD.

Herein, the genomic information of IBD samples was integrated to assess the m^6^A modification patterns. Next, we explored the correlation between the m^6^A modification pattern and immune infiltration, investigating the functions and related mechanism of m^6^A modification patterns in the immune microenvironment in IBD. Surprisingly, we reveal that m^6^A modification patterns are associated with the disease types and infiltration of multiple immune cells. According to differentially expressed genes in different m^6^A modification patterns, we reveal that m^6^A phenotype–related hub genes affect disease characteristics and immune cell-infiltrating characteristics in IBD patients and demonstrate that m^6^A phenotype–related hub genes are connected with disease progression, immune cell infiltration, anti-TNF therapeutic response, and immunotherapy. Assessing the expression of m^6^A phenotype–related hub genes might guide the choice of IBD drugs and improve the prediction of therapeutic response to anti-TNF therapy.

## Materials and Methods

### Inflammatory Bowel Disease Data Set Source and Preprocessing

Public data and full clinical annotation were downloaded from the Gene-Expression Omnibus (GEO) database.^1^ GSE111889 (Lloyd-Price et al., 2019) (251 samples) was obtained in this study for further analysis (Supplementary Table 1). Data preprocessing was performed as follows: the downloaded data set was the expression profile obtained after data homogenization. First, the probes corresponding to the genes were identified, and no-load probes were removed. When multiple probes corresponded to the same gene, the median number was selected as the expression level of the gene. Four samples with zero expression of all the genes were filtered out, and the final expression matrix of 247 samples was used for subsequent analysis. Then, based on gene expression spectrum data and protein-coding genes from the Ensembl database,^2^ 55,765 target gene expression profile data was obtained. Finally, 27 m^6^A methylase regulators (i.e., writers, erasers and readers) in three types were used with reference to previous studies (Deng et al., 2018). Among them, 24 m^6^A methylase regulators were supported by expression profile data (Supplementary Table 2).

### Sample Classification

Patients with qualitatively different m^6^A modification patterns were classified by using the R package of ConsensusClusterPlus (gaptools). The optimal *k* value was chosen by identifying the inflection point of the sum of the squared error (SSE). The decline slows down after *k* = i, and *k* = i is selected.

### Screening for Differential Genes in m^6^Aclusters

Edger analysis (gaptools) was used to identify differentially expressed genes among m^6^Acluster patterns, and 4745 genes and gene expression profiles of differentially expressed genes among m^6^Acluster patterns were obtained. Adjusted *P*-value < 0.05 was considered to be the criteria of differentially expressed genes.

### Construction of the Gene Co-expression Network and Identification of the Key Modules

High-connection genes and modules were identified using the R-package “WGCNA.” Each module was constructed by calculating the soft threshold power β. The WGCNA algorithm was used to construct the co-expression module after setting the soft threshold power value. Adjacency information was converted into topological overlap, and the network connectivity of genes was measured. Based on the dissimilarity of the topological overlap matrix (TOM), a hierarchical clustering function was performed to classify genes with similar expression profiles into modules (the minimum size of gene dendrogram = 25; merged highly similar modules with height = 0.25). The correlation between the clinical traits and the module eigengenes was used to determine the relevant modules. The correlation of gene expression profiles and the module eigengenes was used to quantitatively measure module membership. Gene significance was defined as the absolute value of the correlation between genes and clinical traits. Then, relevant modules of high importance for clinical traits were identified (Supplementary Table 3).

### Functional Enrichment Analysis of Genes in the Key Modules

The R package clusterProfiler was used to perform functional enrichment analysis of genes in the key modules. The results of the molecular function (MF), cellular component (CC), and biological functions (BF) in the Gene Ontology (GO) analysis and the enrichment analysis of Kyoto Encyclopedia of Genes and Genomes (KEGG) were extracted.

### Identification of the Hub Genes in the Co-expression Networks

To search for interacting genes, tgenes in the key modules were imported into a search tool (STRING^3^; score >900) to predict the PPI network. We defined interaction network hub genes as a degree of nodes ≥200 and interaction network non-hub genes as a degree <200 in the PPI (Supplementary Table 4). Genes with significance >0.6 and module membership >0.8 in the modules were defined as key genes (Supplementary Table 5). Finally, hub genes identified in the disease-related module and hub genes in PPI were intersected, and 29 disease-related hub genes were finally obtained (Supplementary Table 6).

### Difference Analysis of Immune Infiltration and Therapeutic Effect

The CiberSort algorithm of CiberSort software was performed to calculate the proportion of immune cell infiltration of different types, and a stacking diagram and box plot were drawn for display. The differential expression of genes at the immune examination sites (PD-L1, IDO1, CD86, ICOS, TNFRSF9, and CTLA4) and the response to anti-TNF treatment were analyzed.

### Statistical Analysis

Most analyses were performed using R software,^4^ Prism software,^5^ and SPSS software^6^ using two-tailed unpaired Student’s *t*-test or log-rank test unless otherwise specified. The chi-square test was applied to compare the response rates to therapy. In all analyses, *P*-values were bilateral, and *P* < 0.05 was considered statistically significant.

## Results

### Landscape of m^6^A Regulators and the Mucosal Immune Microenvironment in IBD

**FIGURE 1:**
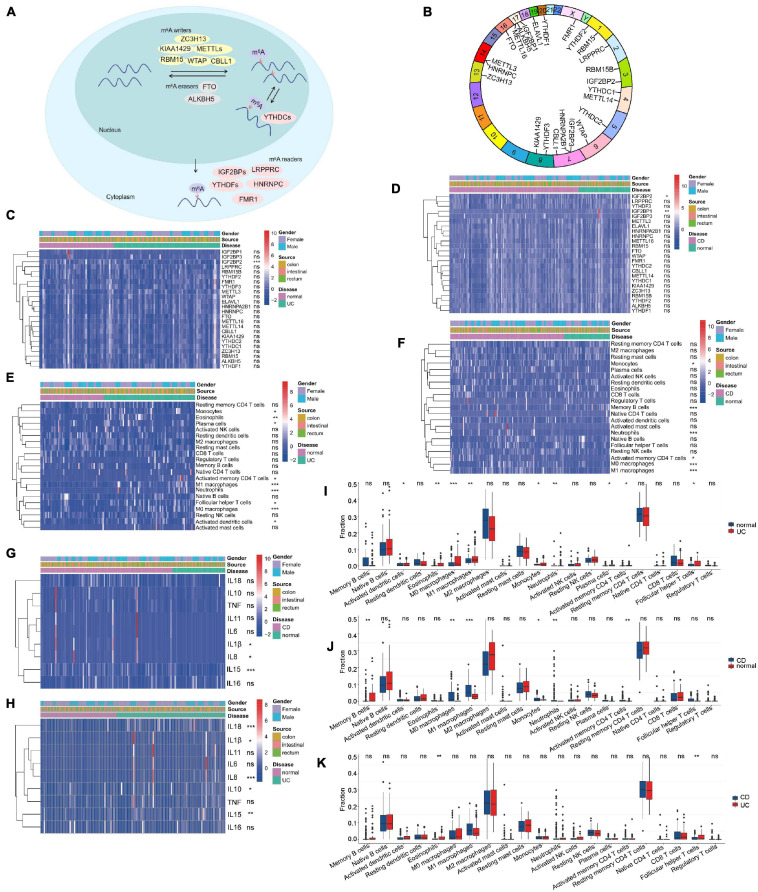
Landscape of m^6^A regulators and immune microenvironment in inflammatory bowel disease (IBD). **(A)** Summary of the reversible process of m^6^A regulator–mediated methylation. **(B)** The location of m^6^A regulators on 24 chromosomes using GSE111889 cohort. **(C,D)** The expression of 24 m^6^A regulators among ulcerative colitis (UC) panel **(C)**, Crohn’s disease (CD) panel **(D)**, and normal tissues. **(E,F,I–K)** The difference of immune cell infiltration among UC, CD, and normal tissues. **(G,H)** The expression of inflammatory factors among normal, UC panel **(H)**, and CD tissues panel **(G)**. The asterisks represented the statistical *P*-value (*P < 0.05; **P < 0.01; ***P < 0.001).

Gene-Expression Omnibus data sets with clinical information and available data (GSE111889, Supplementary Table 1; Lloyd-Price et al., 2019) were enrolled in our study. Based on the previous data (Deng et al., 2018), we identified 24 m^6^A regulators, including nine “writers,” two “erasers,” and 13 “readers” (Supplementary Table 2). The reversible process of m^6^A regulator–mediated methylation is summarized in Figure 1A, and the locations of m^6^A regulators on chromosomes are shown in Figure 1B. To ascertain whether the above m^6^A regulators influenced the disease progression of IBD, we evaluated the mRNA expression of m^6^A regulators in UC, CD, and normal tissues and found that expression of “readers” (i.e., IGF2BP1 and IGF2BP2) was dramatically decreased in CD tissues compared with normal tissues (*P* < 0.01) (Figure 1D). The expression of IGF2BP2 was markedly decreased in UC tissues compared with normal tissues (*P* < 0.001) (Figure 1C). The expression of IGF2BP1, METTL16, YTHDC2, and KIAA1429 was downregulated in CD tissues compared with UC tissues (*P* < 0.05) (Supplementary Figure 1A). These results suggest that m^6^A regulators exhibit high heterogeneity of the expressional alteration landscape among normal, UC, or CD patients, indicating that different m^6^A regulator expression may affect disease progression in different IBD types.

Additionally, we analyzed immune cell-infiltrating characteristics in the intestinal mucosa in IBD and found that UC was characterized by M1 macrophages (*P* < 0.001); M0 macrophages (*P* < 0.001); neutrophils (*P* < 0.001); eosinophils (*P* < 0.01) (Figures 1E,I); and the inflammatory factors IL18, IL8, and IL15 (Figure 1H). M1 macrophages, neutrophils, and M0 macrophages were enriched, but memory B cells were decreased in CD tissues (Figures 1F,J; *P* < 0.001) with the upregulation of IL15, IL8, and IL1β expression (Figure 1G). Compared with CD tissues, eosinophils and follicular helper T cells were more abundant in UC tissues (Figure 1K; *P* < 0.01). These results indicate that alterations in the mucosal immune microenvironment may be the prominent pathogenic factors of IBD, and local infiltration of M1 macrophages and neutrophils may promote the progression of IBD.

### m^6^A Methylation Modification Patterns Are Mediated by 24 m^6^A Regulators in IBD Patients

**FIGURE 2:**
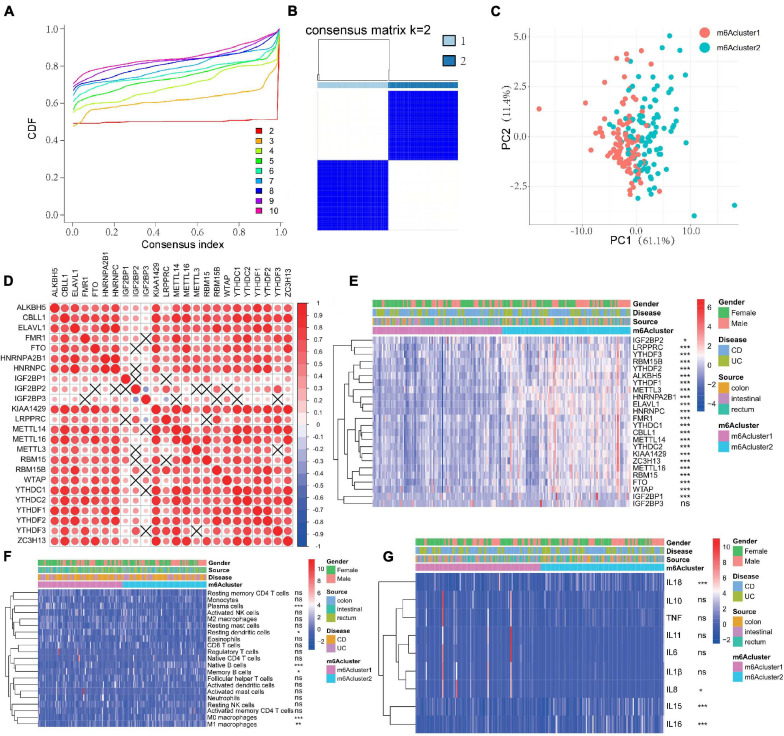
m^6^A methylation modification patterns are correlated with the clinical characteristics and the mucosal immune microenvironment in IBD. **(A–C)** Consensus clustering identified two subgroups according to the expression of m^6^A regulators. **(D)** Evaluation the pairwise correlations among 24 m^6^A regulators’ expression. **(E)** The expression of 24 m^6^A regulators in different m^6^A subtypes and disease subtypes. **(F,G)** The difference of mucosal immune cells infiltration panel **(F)** and inflammatory factors panel **(G)** between m^6^Acluster1 and m^6^Acluster2. The asterisks represented the statistical *P*-value (*P < 0.05; **P < 0.01; ***P < 0.001).

According to the expression of the 24 m^6^A regulators, m^6^A modification patterns were classified using the R package of ConsensusClusterPlus (gaptools). The optimal clustering stability (*k* = 2–10) was determined by the similarity of m^6^A regulator expression and the proportion of ambiguous clustering measurement, and *k* = 2 was identified (Figures 2A,B and Supplementary Figure 1B). Based on unsupervised clustering, we eventually identified two distinct modification patterns, including 99 cases in m^6^Acluster1 and 98 cases in m^6^Acluster2 (Supplementary Table 7). IBD samples could be completely distinguished based on the expression of these 24 m^6^A regulators (Figure 2C). To explore the correlation among “writers,” “erasers,” and “readers,” we evaluated pairwise correlations among the expression of 24 m^6^A regulators in IBD and revealed that positive correlations were more frequent (Figure 2D). We found that remarkable correlations existed among “writers,” “erasers,” and “readers,” and m^6^A regulators with the same function exhibited significant correlations in expression. IBD with high expression of IGF2BP2 (a “reader” gene) exhibited downregulation of IGF2BP3 (a “reader” gene) although upregulation of IGF2BP2 did not affect expression of other genes except for YTHDF3, LRPPRC, and RBM15B (“writer” genes). The “writer” genes CBLL1, KIAA1429, ZC3H13, and METTL16 presented a common trend in FTO (an “eraser” gene) expression. IBD with upregulation of the “writer” gene KIAA1429 displayed a common trend in ALKBH5 (an “eraser” gene) expression. Additionally, the change of IGF2BPs expression did not affect expression of these “eraser” genes. Therefore, crosstalk among different m^6^A regulators may be crucial for the generation of m^6^A modification patterns in individual IBD patients. After unsupervised clustering, we investigated the expression of 24 m^6^A regulators in different m^6^A subtypes and disease subtypes (Figure 2E) and found that the expression of m^6^A methylation regulators was increased in the m^6^Acluster2 compared with the m^6^Acluster1 except for IGF2BP3 (*P* < 0.05). Next, the clinicopathological features between the two subtypes were compared. However, there were no significant differences between m^6^Acluster1 and m^6^Acluster2 with respect to patient gender, location, or disease subtypes (*P* > 0.05).

### m^6^A Modification Patterns Regulate the Mucosal Immune Microenvironment in IBD

We next compared differences in immune cell infiltration between the two m^6^A modification patterns using CIBERSORT. To our surprise, m^6^Acluster2 was prominently associated with immune activation (Figure 2F). Subsequent analyses of immune infiltration suggested that m^6^Acluster2 was significantly abundant in innate immune cell infiltration, including M0 macrophage (*P* < 0.001), M1 macrophage (*P* < 0.01), and acquired immune cells (native B cells, memory B cells; *P* < 0.05). m^6^Acluster1 was enriched in innate immune cell infiltration, including resting DCs (*P* < 0.05) and plasma cells (*P* < 0.001) (Figure 2F). Furthermore, we found that expression of IL15, IL16, and IL18 was upregulated in m^6^Acluster2 (*P* < 0.001). However, expression of IL8 in m^6^Acluster1 was increased (*P* < 0.05) (Figure 2G). The significant differences in the infiltration of immune cells and inflammatory factors between the two m^6^Aclusters indicate that m^6^A methylation modifications may change the mucosal immune microenvironment of IBD.

### Biological Characteristics of Key m^6^A Modules

**FIGURE 3:**
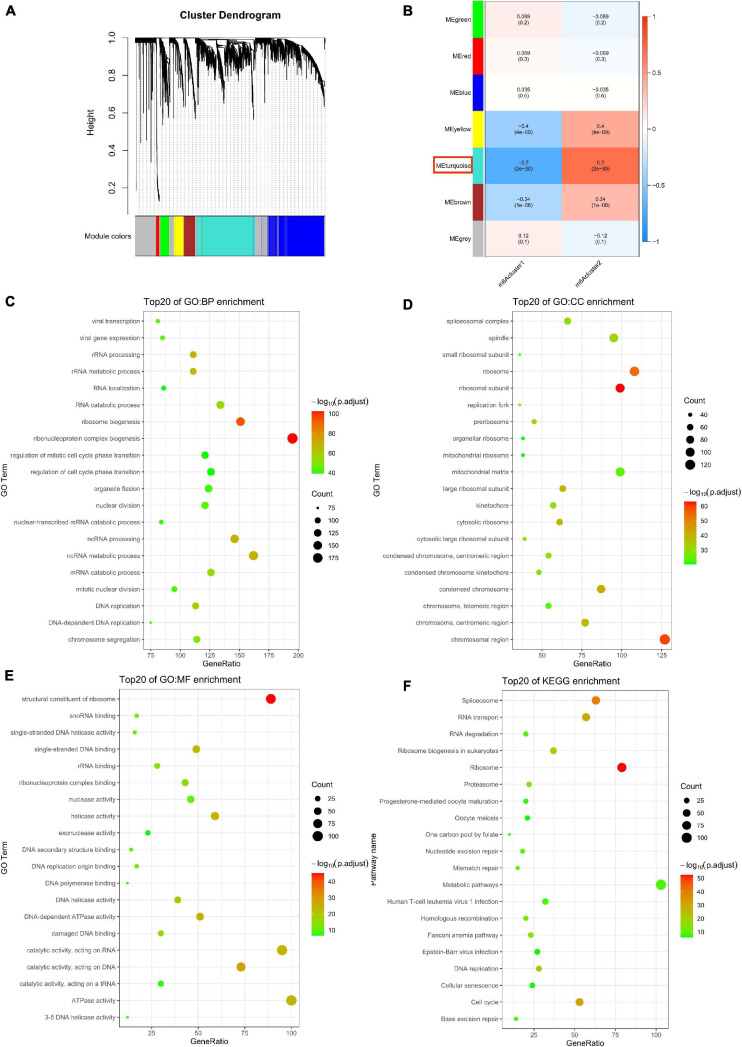
Biological characteristics of key m^6^A module. **(A)** Gene dendrogram obtained by average linkage hierarchical clustering. **(B)** The correlation between the module eigengenes and m^6^A modification patterns. **(C–F)** Functional annotation of the genes in the key m^6^A module by using Gene Ontology (GO) terms of biological processes (BP) panel **(C)**, cellular component (CC) panel **(D)**, molecular function (MF) panel **(E)**, and Kyoto Encyclopedia of Genes and Genomes (KEGG) pathway **(F)**.

To explore the differentially expressed genes in the two m^6^Aclusters, we used the Edger package in GAPTools. Subsequently, 4737 differentially expressed genes were screened out between the two clusters, among which 2274 genes were upregulated and 2463 genes were downregulated. A weighted gene co-expression network was then constructed (Supplementary Figure 1C). After calculating the eigengenes of each module, performing cluster analysis on the modules, and merging the modules close to each other into new modules, we obtained seven modules (Figures 3A,B). The turquoise module was the most correlated with the m^6^Acluster classification phenotype (Figure 3B and Supplementary Table 3; *P* = 2e–30). The R package clusterProfiler was used to analyze the function and pathway information of the top 20 enriched genes in the turquoise module. As shown in Figure 3C, genes in the turquoise module were markedly enriched in the biological processes (BP), including ribonucleoprotein complex biogenesis and ribosome biogenesis (Figure 3C; *P* < 0.05); CC were involved, including the composition of ribosomal subunit, chromosomal region (Figure 3D; *P* < 0.05); MF-related genes were markedly enriched in structural constituent of ribosome (Figure 3E; *P* < 0.05); the KEGG pathway was enriched in ribosome and spliceosome (Figure 3F; *P* < 0.05).

### Twenty-Nine m^6^A Phenotype-Related Hub Genes Are Correlated With the IBD Characteristics

**FIGURE 4:**
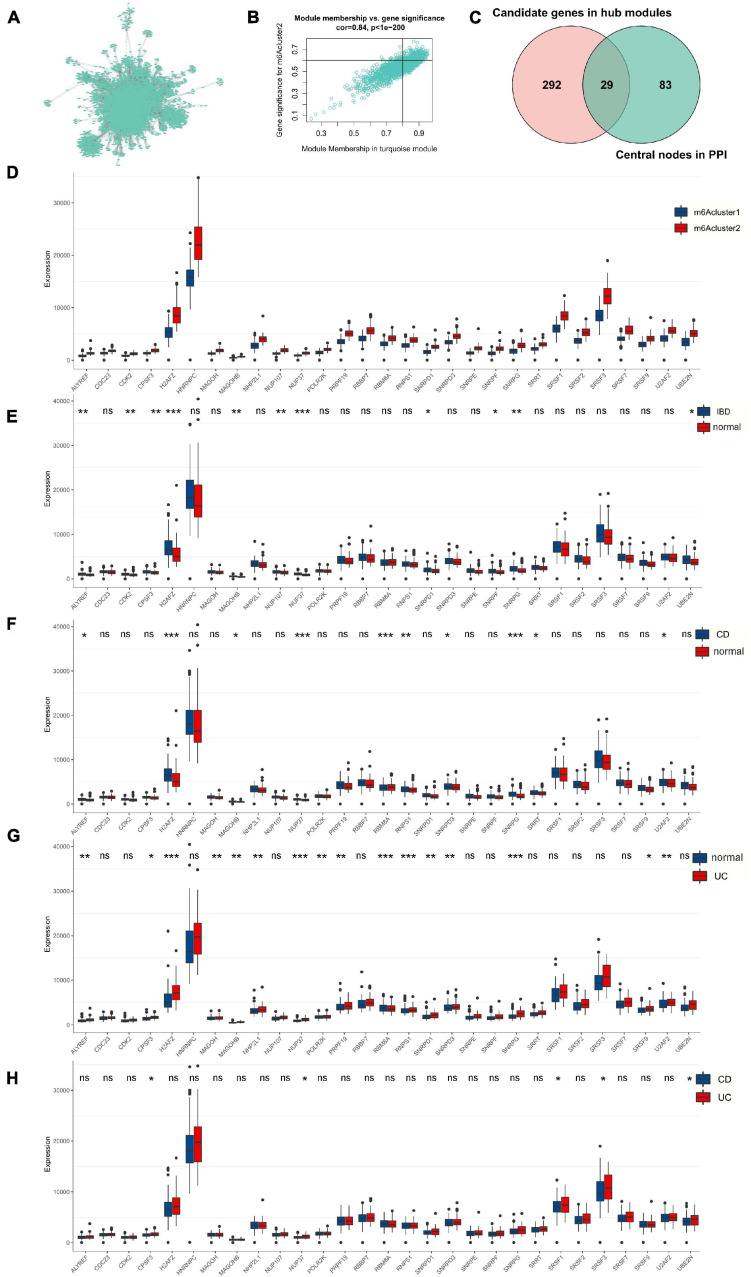
Twenty-nine m^6^A phenotype-related hub genes affect disease characteristics in IBD. **(A)** The PPI network of genes in the key module. **(B)** The significance genes in the turquoise module. **(C)** The hub genes of the interaction network are intersected with the hub genes identified in the m^6^A-related module. **(D)** The 29 genes’ expression in different m^6^Acluster classification. **(E–H)** The 29 genes’ expression between normal, UC, and CD tissues. The asterisks represented the statistical *P*-value (*P < 0.05; **P < 0.01; ***P < 0.001).

To further validate the correlation of m^6^A phenotype–related hub genes with disease characteristics, we constructed a PPI network to analyze 1463 related genes in the key module (Figure 4A), identifying 112 hub genes (Supplementary Table 4). Genes (gene significance >0.6; module membership >0.8) were identified from the turquoise module as key genes with a total of 321 genes (Figure 4B and Supplementary Table 5). Finally, the hub genes of the interaction network were intersected with the hub genes identified in the m^6^A-related module, and 29 disease-related hub genes were obtained (Figure 4C and Supplementary Table 6).

We found that 29 disease-related genes were significantly different among the m^6^Acluster classifications. The expression of 29 genes in m^6^Acluster2 was higher than that in m^6^Acluster1 (Figure 4D; *P* < 0.001). Furthermore, we explored differences in the expression of 29 disease-related genes between normal, CD, and UC tissues and found that the expression of NUP37, SNRPG, H2AFZ, MAGOHB, NUP107, ALYREF, CDK2, F3, SNRPD1, SNRPF, and UBE2N was significantly upregulated in the IBD tissues compared with normal tissues (Figure 4E; *P* < 0.05) although there was no significant difference in the other 18 genes (Figure 4E; *P* > 0.05). Compared with normal tissues, NUP37, H2AFZ, SNRPG, RBM8A, RNPS1, SNRPD3, ALYREF, MAGOHB, U2AF2, and SRRT were increased in CD tissues (Figure 4F; *P* < 0.05). Expression of NUP37, RBM8A, RNPS1, H2AFZ, SNRPG, PRPF19, U2AF2, ALYREF, MAGOHB, NHP2L1, SNRPD1, SNRPD3, MAGOH, POLR2K, CPSF3, and SRSF9 was markedly higher in UC tissues than in normal tissues (Figure 4G; *P* < 0.05). However, only CPSF3, NUP37, SRSF3, UBE2N, and SRSF1 were upregulated in UC tissues but not in CD tissues (Figure 4H; *P* < 0.05). These results suggest that 29 m^6^A phenotype–related hub genes are differentially expressed in different IBD types.

### Consensus Clustering for m^6^A Phenotype–Related Hub Genes With the Characteristics of IBD Patients

**FIGURE 5:**
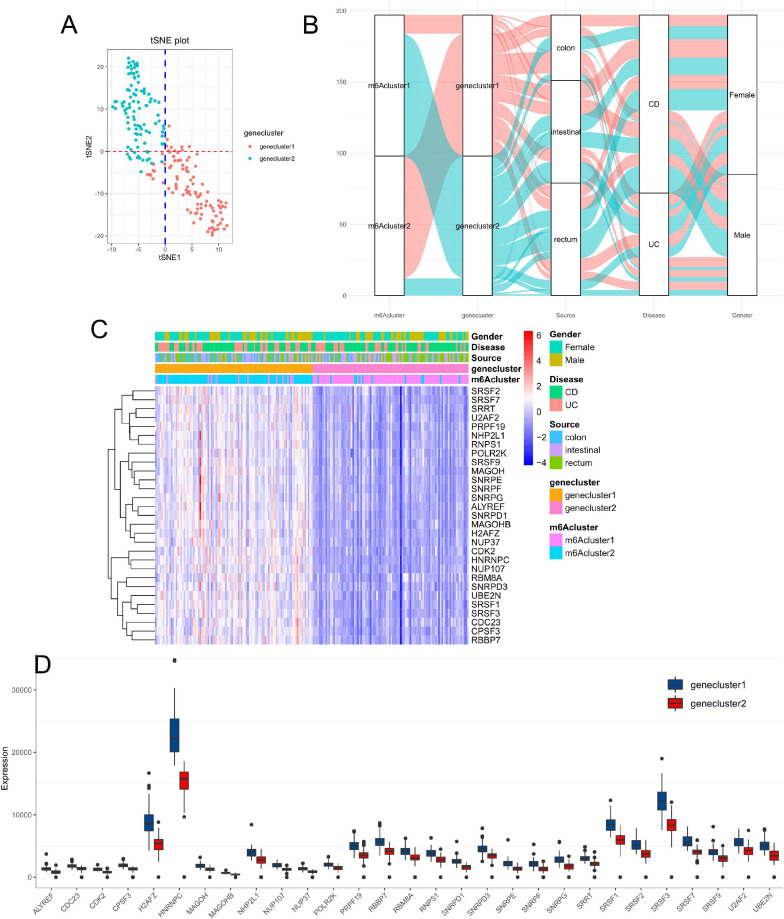
Consensus clustering for m^6^A phenotype–related hub genes with the characteristics of IBD patients. **(A)** Clustering the samples with the tSNE method. **(B)** Alluvial diagram showing the changes of m^6^Aclusters, disease subtype, location, gender, and gene clusters. **(C,D)** The expression of 29 hub genes between genecluster1 and genecluster2.

Based on expression of the 29 m^6^A phenotype-related genes, we classified IBD patients into different genomic subtypes by unsupervised clustering analyses (K-means) and found that the gene clusters could be well distinguished (Figure 5A and Supplementary Figure 1D). We found that there were marked differences among the 29 key disease genes in the gene cluster classification (*P* < 0.001) with higher gene expression in genecluster1 and lower gene expression in genecluster2 (Figure 5C). As shown in Figure 5B, almost all patients with the m^6^Acluster2 subtype were classified into genecluster1. However, no significant difference in patient gender, location, or disease subtypes was observed between genecluster1 and genecluster2 (Figures 5B,C; *P* > 0.05). The expression of all 29 m^6^A phenotype–related hub genes was significantly higher in genecluster1 than in genecluster2 (Figure 5D; *P* < 0.001).

### m^6^A Phenotype–Related Hub Genes Influence Immune Infiltration and Therapeutic Response

**FIGURE 6:**
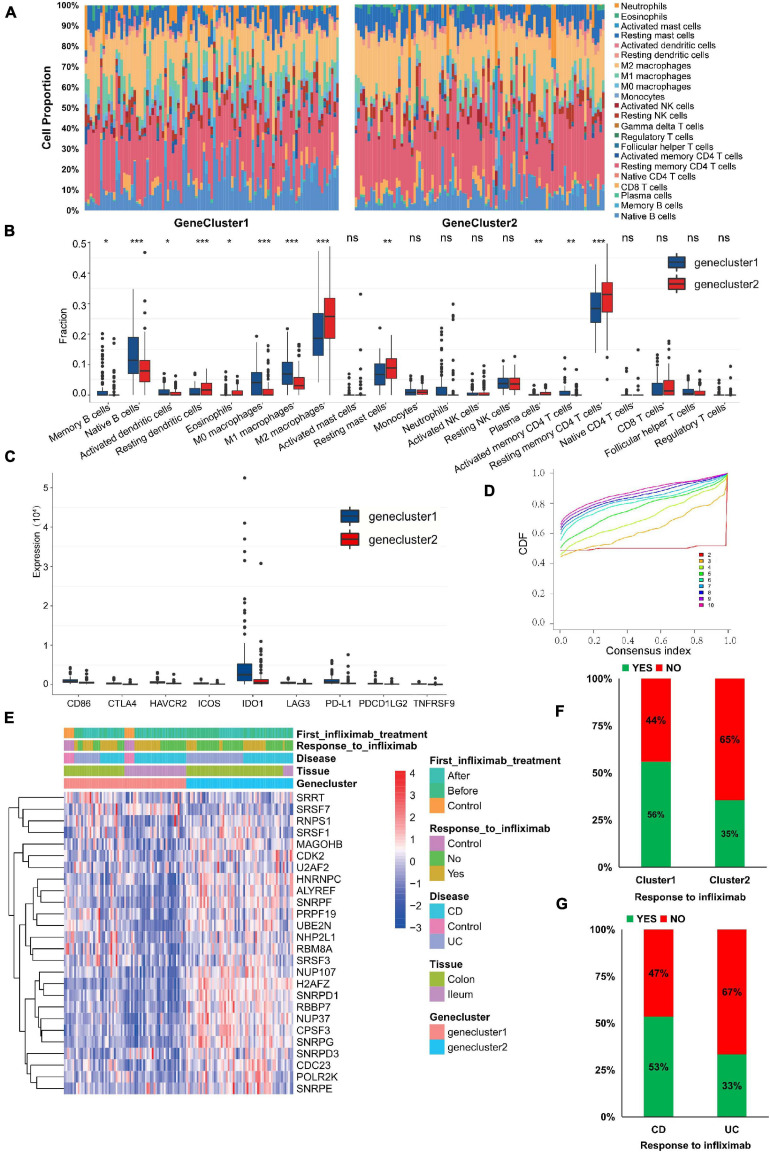
m^6^A phenotype–related hub genes influence the immune infiltration and therapeutic response. **(A,B)** The immune cell infiltrating levels in different gene cluster subtypes in the GSE111889 cohort. **(C)** The expression differences of nine important immune checkpoints among different gene clusters. **(D)** Consensus clustering cumulative distribution function (CDF) for *k* = 2–10. **(E)** The expression of hub genes, anti-TNF therapeutic response, and disease subtype between genecluster1 and genecluster2. **(F,G)** Infliximab therapeutic response in different gene clusters **(F)** and disease subtypes **(G)**. The asterisks represented the statistical *P*-value (*P < 0.05; **P < 0.01; ***P < 0.001).

To explore the effect of m^6^A phenotype–related hub genes on the immune infiltration of IBD, we evaluated immune infiltration between genecluster1 with upregulated m^6^A phenotype-related hub gene expression and genecluster2 with downregulated m^6^A phenotype-related hub gene expression. Genecluster1 displayed higher infiltration levels of M1 macrophages, M0 macrophages, naive B cells, CD4 memory-activated T cells, memory B cells, and activated DCs, whereas genecluster2 was more correlated with resting DCs, M2 macrophages, CD4 memory resting T cells, plasma cells, resting mast cells, and eosinophils (Figures 6A,B; *P* < 0.05).

Expression differences of nine important immune checkpoints supported by expression data among different gene clusters are shown in Figure 6C. We found that the expression of all immune checkpoints in genecluster1 was significantly higher than that in genecluster2 (*P* < 0.01), indicating that m^6^A phenotype–related hub genes might affect immunotherapy. Current treatments are biological agents anti-TNF (primarily infliximab) for refractory and severe forms of IBD. Thus, we further verified the 29 m^6^A phenotype–related hub genes in GSE16879 (Arijs et al., 2009), which was available data and clinical treatment information. However, the expression of only 26 hub genes was supported by expression data in the GSE16879 data set. Based on the expression of 26 hub genes, we classified patients using the R package of ConsensusClusterPlus (gaptools), and *k* = 2 was identified (Figure 6D and Supplementary Figure 1E). Significant differences in infliximab therapy existed between the two gene clusters (Figure 6E). The expression of hub genes (H2AFZ, NUP37, SNRPD1, CPSF3, RBBP7, and SNRPG) in genecluster2 was significantly higher than in genecluster1, whereas expression of SRSF7 and SRRT in genecluster2 were lower than in genecluster1 (Figure 6E; *P* < 0.05). The response to infliximab in genecluster1 was better than that in genecluster2 (Figure 6F), and the response to infliximab in CD was better than that in UC (Figure 6G), suggesting that m^6^A-related hub gene expression may affect the therapeutic effect of infliximab. These results indicate that IBD with high expression of some m^6^A phenotype–related hub genes may represent those characterized by resistance to infliximab. Assessing the expression of m^6^A phenotype–related hub genes might guide the choice of IBD drugs and predict the response to anti-TNF therapy.

## Discussion

Currently, as most studies on disease-related m^6^A modification are limited to various tumors, single regulators, or single immune cell types, the effects of multiple m^6^A regulators on the overall immune infiltration characteristics and on IBD progression are poorly understood. Clarifying the role of m^6^A modification in the immune cell infiltration of IBD will improve our understanding of disease progression and disease-related local immune infiltration and strengthen more effective therapeutic strategies.

N6-methyladenosine regulators are involved in inflammatory development. However, only one study reports an association between m^6^A regulators and disease progression in IBD, and it reports that METTL14 (a “writer” gene) deficiency in T cells promotes spontaneous colitis in mice (Lu et al., 2020). IGF2BPs, a new family of m^6^A “readers,” are composed of two RNA recognition motif domains and four K homology domains (Bell et al., 2013) and promote the processes of various tumors (Huang X. et al., 2018; Huang S. et al., 2019; Hanniford et al., 2020). MYC, ACTIN, and LIN28B are reported as targets of IGF2BPs (Nielsen et al., 1999). Here, consistent with a previous study (Chen et al., 2021), we find that not only remarkable correlations exist among “writers,” “erasers,” and “readers,” but also that m^6^A regulators with the same function display significant correlations in expression. We find that expression of “readers” (i.e., IGF2BP1 and IGF2BP2) was downregulated in CD tissues compared with normal tissues, and expression of the IGF2BP2 was markedly decreased in UC tissues. The m^6^A regulators among normal, UC, and CD patients exhibit high heterogeneity of the expressional alteration landscape. However, the exact molecular mechanisms by which m^6^A regulators modulate IBD (UC and CD) development remain elusive.

As a reversible RNA methylation, m^6^A is added by “writers” and removed by “erasers,” indicating that they may represent opposite roles (Lan et al., 2019). However, it is interesting to find that m^6^A writers and erasers were positively correlated in our study. The “writer” genes CBLL1, KIAA1429, ZC3H13, and METTL16 presented a common trend in FTO (an “eraser” gene) expression. The “writer” gene KIAA1429 displayed a common trend in ALKBH5 (an “eraser” gene) expression in IBD. Previous studies find that m^6^A writers that increase the RNA m^6^A level are oncogenes (Lan et al., 2019; Qian et al., 2019; Wang X. K. et al., 2021), and FTO and ALKBH5 that decrease the RNA m^6^A level are also oncogenes (Shen et al., 2020; Su et al., 2020). The targets of “erasers” and “writers” that are reported in different studies are different, suggesting different mechanisms and other regulatory factors exist in disease development other than the “writer” and “eraser.” Additionally, the m^6^A “readers” exert post-transcriptional functions, which may partly account for the seemingly contradictory roles between the “eraser” and “writer.” In the future, the effect of m^6^A “writers” and “erasers” on the same RNA simultaneously should be examined to explore whether other regulatory factors are involved in m6A modification.

More recently, m^6^A regulators are shown to play crucial roles in disease-related immune cell infiltration. ALKBH5 inhibits antiviral innate responses by erasing their m^6^A modification (Zheng et al., 2017). METTL3, potentially serving as an anti-inflammatory target, methylates STAT1 mRNA and drives M1 macrophage polarization (Liu et al., 2019; Wang J. et al., 2019). LPS-induced inflammatory responses are regulated by the m^6^A reader YTHDF2 in macrophages (Yu et al., 2019). FTO knockdown induces a decrease of PPAR-γ and STAT1 transcripts through accelerating mRNA degradation mediated by YTHDF and inhibiting M1/M2 activation (Gu et al., 2020). It is reported that the immune cells contributing to IBD progression are predominantly M1 macrophages, neutrophils, and Th cells (de Souza and Fiocchi, 2016; Zhou et al., 2018, 2019). The m^6^A “writer” protein METTL3 has important roles in reprograming naive T cells for proliferation and differentiation (Li et al., 2017). Deficiency of METTL14, another m^6^A “writer,” causes induction of native T cells into induced Treg cells (Lu et al., 2020). In this study, we find that m^6^Acluster2 with a higher expression of m^6^A regulators was significantly abundant in innate immune cell infiltration, including M0 macrophage, M1 macrophage, and acquired immune cells (native B cells and memory B cells), and m^6^Acluster1 with a lower expression of m^6^A regulators was enriched in innate immune cell infiltration, including resting DCs and acquired immune cells (plasma cells), suggesting that m^6^A methylation modification may upregulate innate immune cells associated with inflammation and inhibit the activation of acquired immune B cells during IBD disease progression. In addition, neutrophils and activated memory CD4 T cells were more highly expressed in CD and UC tissues than in normal tissues, but no significant differences in the T cell types or neutrophils exist between the two m^6^A modification patterns, indicating that m^6^A regulators may influence inflammation development primarily by recruiting macrophages. METTL3 deficiency increases the secretion of IL8 and elevates the recruitment of neutrophils (He et al., 2021). In our study, IL8 expression in m^6^Acluster1 was increased, and expression of IL15, IL16, and IL18 was upregulated in m^6^Acluster2, suggesting that m^6^A methylation modification may change mucosal inflammatory factors in IBD. Nevertheless, our data on m^6^A regulators are confined to bioinformatics analysis, and more research is needed to explore the exact molecular mechanisms by which these disease-related m^6^A regulators induce immune cell infiltration in IBD.

Next, we identified 29 disease-related hub genes from the key module, which was the most correlated with the m^6^Acluster phenotype, and found that expression of NUP37, SNRPG, H2AFZ, and ALYREF was significantly upregulated in the IBD (CD and UC) tissues compared with normal tissues. NUP37, a component of the nuclear pore complex, is both a significantly mutated gene and a tumor-destructive gene in various carcinomas (Chen J. et al., 2019; Huang L. et al., 2020). SNRPG belongs to the small nuclear ribonucleoprotein peptide family, which might participate in tumor chemotherapeutic resistance (Lan et al., 2020). H2AFZ is significantly upregulated in hepatocellular carcinoma and is related to poor prognosis (Tang et al., 2020). ALYREF activates the Wnt/β-catenin pathway to promote cell proliferation (Wang J. et al., 2021). However, there are few reports on the association of these genes with IBD or other inflammatory diseases. Our study suggests that these hub genes may be downstream targets of m^6^A regulators and can be expected to trigger new therapeutic strategies for IBD. However, further experiments are needed to verify their roles in IBD development.

Furthermore, we classified patients into different genomic subtypes according to expression of the 29 obtained m^6^A phenotype–related hub genes. Genecluster1, with higher expression of m^6^A phenotype-related hub genes, exhibited higher infiltration levels of M1 macrophages, M0 macrophages, naive B cells, CD4 memory-activated T cells, memory B cells, and activated DCs, whereas genecluster2 was more correlated with resting DCs, M2 macrophages, CD4 memory resting T cells, plasma cells, resting mast cells, and eosinophils. Despite these findings, these hub genes could, at least in part, explain how m^6^A regulators stimulate the inflammation-associated immune cell infiltration in IBD.

N6-methyladenosine regulators are closely associated with immunotherapy (Yang et al., 2019; Li et al., 2020). For example, inhibiting FTO sensitizes melanoma cells to anti-PD-1 and interferon gamma (IFNγ) treatment (Yang et al., 2019). Knockout of YTHDF1 enhances the therapeutic efficacy of PD-L1 checkpoint blockade in mice (Han et al., 2019). Downregulation of ALKBH5 correlates with a positive response to PD-1 blockade in melanoma patients (Li et al., 2020). In this study, we found that expression levels of all immune checkpoints in genecluster1 with a higher expression of m^6^A phenotype-related hub genes was significantly increased, suggesting that m^6^A phenotype–related hub genes might influence immunotherapy. Furthermore, as current treatments are mostly the biological agent anti-TNF (primarily infliximab) for refractory and severe forms of IBD (Koelink et al., 2020), we verified the m^6^A phenotype–related hub genes in GSE16879 and found that the therapeutic effect of cluster1 with the lower expression of hub genes, including H2AFZ, NUP37, SNRPD1, CPSF3, and RBBP7, was better than that of cluster2. Furthermore, the therapeutic effect in CD was better than that in UC, indicating that IBD with high expression of some m^6^A phenotype-related hub genes (H2AFZ, NUP37, SNRPD1, CPSF3, and RBBP7) may represent those characterized by resistance to infliximab. Our findings provide new possibilities for enhancing the efficacy of anti-TNF therapy for IBD. However, our study is on bioinformatics and is retrospective in nature, and the predictive value of gene signatures should be further verified.

## Conclusion

We identified for the first time the alterations and correlations among m^6^A modulators, m^6^A-related immune infiltration, m^6^A phenotype–related hub genes, and therapeutic response in IBD. Assessing the expression of m^6^A phenotype–related hub genes may guide the choice of IBD drugs and improve the prediction of therapeutic response to anti-TNF therapy.

## Data Availability Statement

The datasets presented in this study can be found in online repositories. The names of the repository/repositories and accession number(s) can be found in the article/Supplementary Material.

## Ethics Statement

The IBD patient data in our study were downloaded from the publicly available data sets in which informed consent were complete.

## Author Contributions

SH and YC: study design and drafting manuscript. YC and JL: data analysis and statistical analysis. All authors contributed to the article and approved the submitted version.

## Conflict of Interest

The authors declare that the research was conducted in the absence of any commercial or financial relationships that could be construed as a potential conflict of interest.

## Publisher’s Note

All claims expressed in this article are solely those of the authors and do not necessarily represent those of their affiliated organizations, or those of the publisher, the editors and the reviewers. Any product that may be evaluated in this article, or claim that may be made by its manufacturer, is not guaranteed or endorsed by the publisher.

## Footnotes

1
https://www.ncbi.nlm.nih.gov/gds

2
https://grch37.ensembl.org/index.html

3
https://string-db.org/

4
https://www.bioconductor.org/

5
https://www.graphpad.com/

6
https://www.ibm.com/analytics/spss-statistics-software

## Supplementary Material

Supplementary Figure 1 Landscape of m^6^A regulators in IBD subtypes and consensus clustering and gene co-expression network in different IBD cohort. **(A)** The expression of 24 m^6^A regulators between UC and CD tissues. **(B)** Consensus clustering CDF for *k* = 2–10 based on m^6^A regulators’ expression in the GSE111889 cohort. **(C)** A weighted gene co-expression network is constructed for candidate gene sets. **(D)** Clustering CDF for *k* = 2–10 based on the expression of hub genes in the GSE111889 cohort. **(E)** Consensus clustering CDF for *k* = 2–10 based on hub genes expression in the GSE16879 cohort. The asterisks represented the statistical *P*-value (^∗^*P* < 0.05).
