# Supplementary figures and images for: m6A Modification Mediates Mucosal Immune Microenvironment and Therapeutic Response in Inflammatory Bowel Disease
Source: Front Cell Dev Biol. 2021 Aug 6;9:692160. doi: 10.3389/fcell.2021.692160 (PMC8378837; doi:10.3389/fcell.2021.692160)

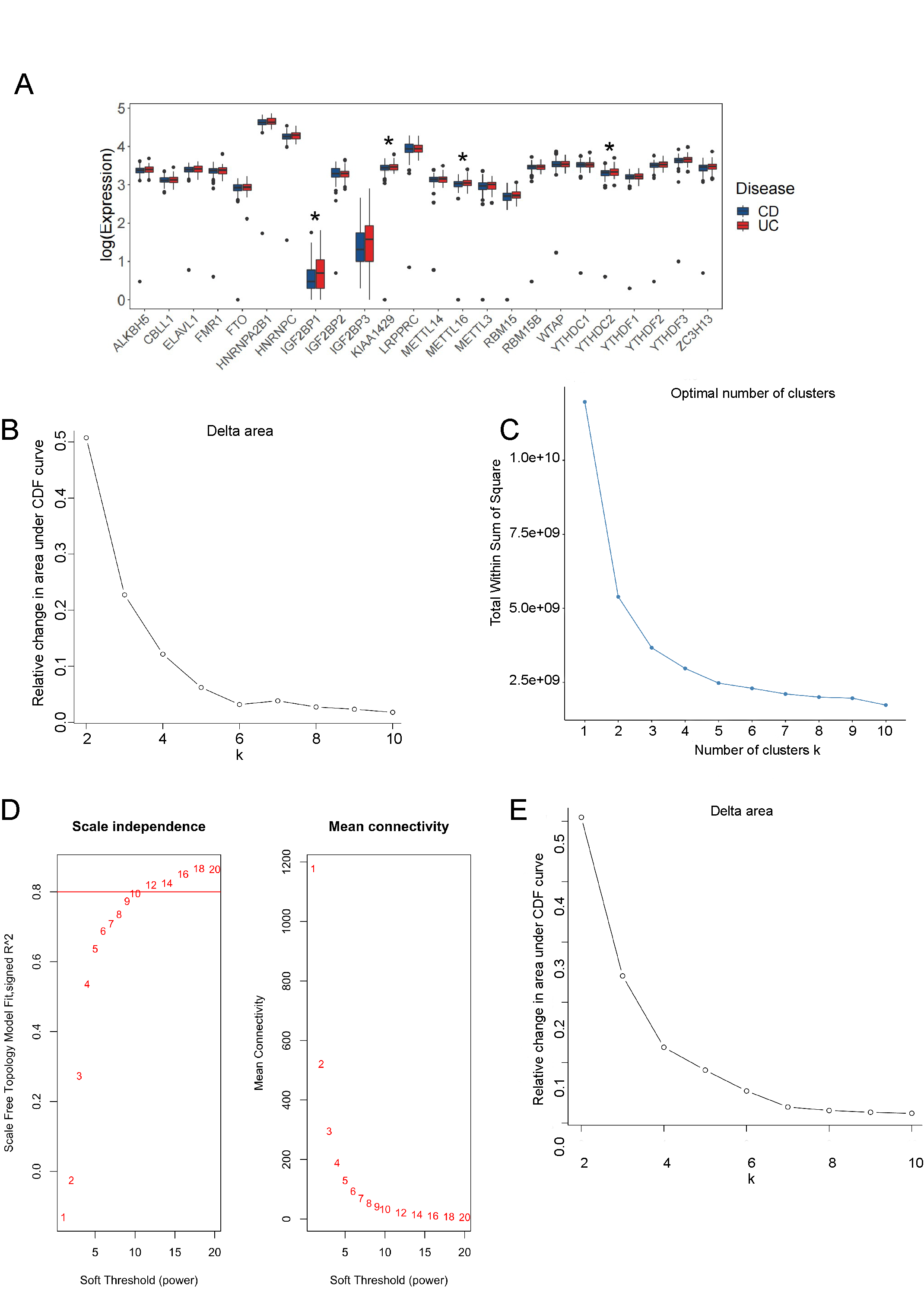

Supplement: Supplementary Figure 1 — Landscape of m6A regulators in IBD subtypes and consensus clustering and gene co-expression network in different IBD cohort. (A) The expression of 24 m6A regulators between UC and CD tissues. (B) Consensus clustering CDF for k = 2–10 based on m6A regulators’ expression in the GSE111889 cohort. (C) A weighted gene co-expression network is constructed for candidate gene sets. (D) Clustering CDF for k = 2–10 based on the expression of hub genes in the GSE111889 cohort. (E) Consensus clustering CDF for k = 2–10 based on hub genes expression in the GSE16879 cohort. The asterisks represented the statistical P-value (∗P < 0.05). [file Image_1.TIF]
