# Supplementary material for: m6A Modification Mediates Mucosal Immune Microenvironment and Therapeutic Response in Inflammatory Bowel Disease
Source: Front Cell Dev Biol. 2021 Aug 6;9:692160. doi: 10.3389/fcell.2021.692160 (PMC8378837; doi:10.3389/fcell.2021.692160)
Supplement: Supplementary file 2 [file Table_1.DOC]

Supplementary Table 1. The clinical information of data

| Infomation | Detail |
| --- | --- |
| GEO Datasets | GSE111889 |
| Platform | GPL11154 |
| Location |  |
| Colon | 51 |
| Ileum | 103 |
| Intestinal | 93 |
| Disease subtypes |  |
| Control | 50 |
| UC | 72 |
| CD | 125 |
| Gender |  |
| Female | 138 |
| Male | 109 |
