# Supplementary material for: m6A Modification Mediates Mucosal Immune Microenvironment and Therapeutic Response in Inflammatory Bowel Disease
Source: Front Cell Dev Biol. 2021 Aug 6;9:692160. doi: 10.3389/fcell.2021.692160 (PMC8378837; doi:10.3389/fcell.2021.692160)
Supplement: Supplementary file 3 [file Table_2.DOC]

Supplementary Table 2. 24 m^6^A regulators in this study

| Gene symbol | m^6^A Type |
| --- | --- |
| ZC3H13 | m^6^A writers |
| METTL3 | m^6^A writers |
| METTL14 | m^6^A writers |
| METTL16 | m^6^A writers |
| KIAA1429 | m^6^A writers |
| RBM15 | m^6^A writers |
| RBM15B | m^6^A writers |
| WTAP | m^6^A writers |
| CBLL1 | m^6^A writers |
| HNRNPA2B1 | m^6^A readers |
| HNRNPC | m^6^A readers |
| YTHDC1 | m^6^A readers |
| YTHDC2 | m^6^A readers |
| YTHDF1 | m^6^A readers |
| YTHDF2 | m^6^A readers |
| YTHDF3 | m^6^A readers |
| ELAVL1 | m^6^A readers |
| FMR1 | m^6^A readers |
| IGF2BP1 | m^6^A readers |
| IGF2BP2 | m^6^A readers |
| IGF2BP3 | m^6^A readers |
| LRPPRC | m^6^A readers |
| FTO | m^6^A erasers |
| ALKBH5 | m^6^A erasers |
