# Supplementary material for: m6A Modification Mediates Mucosal Immune Microenvironment and Therapeutic Response in Inflammatory Bowel Disease
Source: Front Cell Dev Biol. 2021 Aug 6;9:692160. doi: 10.3389/fcell.2021.692160 (PMC8378837; doi:10.3389/fcell.2021.692160)
Supplement: Supplementary file 4 [file Table_3.DOC]

Supplementary Table 3. The number of genes in each module

| Module | No. of Genes |
| --- | --- |
| blue | 1348 |
| brown | 295 |
| green | 217 |
| grey | 1100 |
| red | 80 |
| turquoise | 1463 |
| yellow | 234 |
