# Supplementary material for: m6A Modification Mediates Mucosal Immune Microenvironment and Therapeutic Response in Inflammatory Bowel Disease
Source: Front Cell Dev Biol. 2021 Aug 6;9:692160. doi: 10.3389/fcell.2021.692160 (PMC8378837; doi:10.3389/fcell.2021.692160)
Supplement: Supplementary file 5 [file Table_4.DOC]

Supplementary Table 4. The central node information of PPI network

| Gene name | Degree |
| --- | --- |
| RPS27A | 1104 |
| CDK1 | 467 |
| TP53 | 461 |
| CDC20 | 460 |
| POLR2G | 380 |
| PLK1 | 364 |
| EIF4A3 | 353 |
| POLR2D | 352 |
| UBE2N | 352 |
| TCEB1 | 350 |
| UBE2C | 345 |
| TRIM28 | 335 |
| RNPS1 | 325 |
| NHP2L1 | 321 |
| H2AFX | 317 |
| POLR2K | 314 |
| SKP2 | 309 |
| RBM8A | 302 |
| MAGOH | 299 |
| MAGOHB | 297 |
| RPS6 | 294 |
| H2AFZ | 289 |
| RPS2 | 286 |
| CCNB1 | 284 |
| CDC23 | 284 |
| RPS27 | 281 |
| RPS3 | 278 |
| GTF2F2 | 278 |
| HDAC1 | 278 |
| RPS7 | 271 |
| UBE2S | 265 |
| BRCA1 | 263 |
| RBBP7 | 261 |
| POMC | 257 |
| RPS23 | 254 |
| HSP90AA1 | 254 |
| AURKB | 252 |
| UBE2I | 252 |
| RPL11 | 252 |
| RPS19 | 252 |
| RPS13 | 250 |
| RPS3A | 249 |
| RPS5 | 248 |
| SNRPE | 247 |
| RPS25 | 247 |
| RPS28 | 247 |
| RPS8 | 246 |
| SNRPD2 | 244 |
| CCNA2 | 243 |
| RPS20 | 242 |
| SNRPD3 | 241 |
| SNRPG | 241 |
| RPS21 | 241 |
| CCNF | 241 |
| RPSA | 240 |
| RPL5 | 240 |
| RPS29 | 240 |
| ANAPC10 | 240 |
| RPS12 | 239 |
| RPL9 | 239 |
| SNRPF | 238 |
| SNRPB | 238 |
| RPS24 | 238 |
| SRSF1 | 236 |
| PRPF19 | 235 |
| SNRPD1 | 234 |
| ALYREF | 234 |
| RPL23A | 233 |
| RPL23 | 233 |
| U2AF2 | 232 |
| SRSF3 | 228 |
| SRSF7 | 226 |
| SRSF2 | 226 |
| CDK2 | 225 |
| DHX15 | 225 |
| RPL35 | 224 |
| RPL15 | 224 |
| CPSF4 | 222 |
| CPSF3 | 221 |
| HNRNPC | 220 |
| RPL30 | 219 |
| SRRT | 219 |
| RPL19 | 218 |
| SRSF9 | 218 |
| NUP107 | 216 |
| RPL10A | 216 |
| AURKA | 215 |
| RPL38 | 215 |
| RPL4 | 215 |
| RPA2 | 215 |
| RPL18A | 214 |
| NUP37 | 214 |
| DHX9 | 213 |
| RPL32 | 213 |
| RPLP0 | 212 |
| LRR1 | 211 |
| RPL37A | 211 |
| RPL13 | 210 |
| RPL31 | 210 |
| MAD2L1 | 209 |
| KIF2C | 209 |
| RPL24 | 209 |
| RPL27A | 208 |
| RPL6 | 208 |
| RPL36 | 208 |
| RPL27 | 207 |
| BUB1B | 205 |
| EIF4E | 205 |
| NUP43 | 201 |
| RPA3 | 200 |
| RPL34 | 200 |
| RPL35A | 200 |
