# Supplementary material for: m6A Modification Mediates Mucosal Immune Microenvironment and Therapeutic Response in Inflammatory Bowel Disease
Source: Front Cell Dev Biol. 2021 Aug 6;9:692160. doi: 10.3389/fcell.2021.692160 (PMC8378837; doi:10.3389/fcell.2021.692160)
Supplement: Supplementary file 6 [file Table_5.DOC]

Supplementary Table 5. The module membership of turquoise module and gene significance of hub genes in cluster2

| Gene | Gene significance | Module membership |
| --- | --- | --- |
| ACTL6A | 0.676615274 | 0.88840922 |
| ACTR3B | 0.618053614 | 0.865254285 |
| ADSL | 0.635528477 | 0.941635454 |
| ALG8 | 0.61516305 | 0.864874362 |
| ALYREF | 0.610778225 | 0.882706583 |
| ANAPC7 | 0.635538622 | 0.899688844 |
| ANP32B | 0.613467578 | 0.885153505 |
| APEX1 | 0.635330809 | 0.897553012 |
| APTX | 0.62535397 | 0.908001148 |
| ASF1B | 0.610671074 | 0.824894797 |
| ATIC | 0.627842195 | 0.86245376 |
| ATXN10 | 0.614473971 | 0.911453275 |
| BANF1 | 0.615918866 | 0.897803762 |
| BCCIP | 0.670748959 | 0.932647394 |
| BMS1 | 0.615074895 | 0.877289577 |
| BORA | 0.610961023 | 0.809084666 |
| BRIX1 | 0.637491545 | 0.904983146 |
| BUB3 | 0.651577797 | 0.904005205 |
| C1orf112 | 0.644615172 | 0.85579367 |
| C1QBP | 0.60114621 | 0.814285606 |
| C5orf34 | 0.630531921 | 0.814322267 |
| CBWD2 | 0.607943621 | 0.840047582 |
| CBX3 | 0.613840039 | 0.806461723 |
| CCAR1 | 0.640988697 | 0.819746716 |
| CCAR2 | 0.611491577 | 0.89675468 |
| CCDC43 | 0.621694498 | 0.889709985 |
| CCT2 | 0.61498611 | 0.875005568 |
| CCT3 | 0.643495434 | 0.927647399 |
| CCT4 | 0.602344747 | 0.902693098 |
| CCT5 | 0.61135486 | 0.859089557 |
| CCT6A | 0.625950941 | 0.879555061 |
| CCT7 | 0.655499134 | 0.950273855 |
| CCT8 | 0.641507888 | 0.905708097 |
| CDC123 | 0.631993013 | 0.940474871 |
| CDC23 | 0.60809821 | 0.836474811 |
| CDK2 | 0.608776621 | 0.830625059 |
| CDK5RAP1 | 0.613375408 | 0.87734521 |
| CENPH | 0.644320432 | 0.849881886 |
| CENPL | 0.621627955 | 0.823778185 |
| CEP78 | 0.622361091 | 0.815103207 |
| CHAF1A | 0.652131109 | 0.907433242 |
| CHEK2 | 0.658959926 | 0.897502977 |
| CKS1B | 0.620528693 | 0.862697769 |
| CKS2 | 0.605600323 | 0.837282488 |
| CLN6 | 0.646778939 | 0.851579782 |
| CMSS1 | 0.649873671 | 0.891257191 |
| COPS3 | 0.664737049 | 0.947320079 |
| COPS4 | 0.62773866 | 0.88942885 |
| CPSF3 | 0.628416554 | 0.93650412 |
| CPSF6 | 0.672298375 | 0.809129162 |
| CSTF3 | 0.660756345 | 0.907103724 |
| CWC27 | 0.690909279 | 0.905374025 |
| DAP3 | 0.615302893 | 0.937709549 |
| DAZAP1 | 0.635030058 | 0.849158985 |
| DBF4 | 0.627882284 | 0.845583514 |
| DCAF13 | 0.648566444 | 0.866747188 |
| DHODH | 0.602244554 | 0.849980878 |
| DKC1 | 0.666262547 | 0.891899758 |
| DNAJC2 | 0.618738649 | 0.842305222 |
| DNAJC8 | 0.60722878 | 0.889448242 |
| DNAJC9 | 0.681978525 | 0.942679295 |
| DPH2 | 0.608799634 | 0.868357069 |
| DPH5 | 0.602996554 | 0.895379664 |
| DPM1 | 0.660643269 | 0.842109734 |
| DUT | 0.651087565 | 0.887505391 |
| E2F4 | 0.61751212 | 0.872208441 |
| EFTUD2 | 0.638309042 | 0.930338984 |
| EIF2B5 | 0.656048553 | 0.902753519 |
| EIF3M | 0.61025207 | 0.918440898 |
| EIF5B | 0.636090751 | 0.844297092 |
| ELAVL1 | 0.688892303 | 0.953296883 |
| EMC8 | 0.600582243 | 0.866807596 |
| EMG1 | 0.664011416 | 0.924126717 |
| ENOPH1 | 0.65934764 | 0.884269622 |
| ERH | 0.697788242 | 0.882844013 |
| EWSR1 | 0.672681875 | 0.85812426 |
| EXOSC10 | 0.630972292 | 0.909920366 |
| EXOSC2 | 0.651900364 | 0.924150207 |
| EXOSC7 | 0.608661722 | 0.86264324 |
| EXOSC8 | 0.654314998 | 0.899035883 |
| EXOSC9 | 0.635053208 | 0.893755429 |
| EZH2 | 0.63783634 | 0.850167214 |
| FANCD2 | 0.602138601 | 0.802721611 |
| FANCE | 0.650990342 | 0.87515144 |
| FEN1 | 0.613758124 | 0.869575635 |
| FTSJ3 | 0.622492178 | 0.891807401 |
| FXN | 0.616822891 | 0.882007312 |
| GART | 0.604252384 | 0.85769173 |
| GEMIN2 | 0.612399486 | 0.892006009 |
| GGCT | 0.629046665 | 0.819639186 |
| GINS3 | 0.634826104 | 0.865278391 |
| GMPS | 0.627054126 | 0.873237727 |
| GNL2 | 0.600777958 | 0.895940621 |
| GPATCH4 | 0.637128587 | 0.876047152 |
| GPN3 | 0.620506046 | 0.897327913 |
| GTPBP4 | 0.608467495 | 0.860887637 |
| H2AFZ | 0.655212736 | 0.894871067 |
| HAT1 | 0.658265885 | 0.908389566 |
| HAUS1 | 0.628948275 | 0.902157441 |
| HDAC2 | 0.602668739 | 0.864386813 |
| HDAC8 | 0.626344921 | 0.872432274 |
| HMGB1 | 0.682342318 | 0.834122064 |
| HMGN1 | 0.683644912 | 0.819529703 |
| HNRNPA0 | 0.651807413 | 0.872330324 |
| HNRNPA1 | 0.639490789 | 0.854577414 |
| HNRNPA1P48 | 0.645730293 | 0.805945582 |
| HNRNPA2B1 | 0.770355055 | 0.867251938 |
| HNRNPA3 | 0.760395498 | 0.890031721 |
| HNRNPC | 0.684325131 | 0.895056917 |
| HNRNPF | 0.651157025 | 0.870730061 |
| HNRNPK | 0.681618593 | 0.85641548 |
| HNRNPL | 0.690631163 | 0.853104598 |
| HNRNPM | 0.684002873 | 0.940154731 |
| HNRNPR | 0.715637412 | 0.841634666 |
| ILF2 | 0.648672526 | 0.928154793 |
| ILF3 | 0.630196745 | 0.823661744 |
| INTS8 | 0.609123327 | 0.836144416 |
| IPO9 | 0.601239243 | 0.851025256 |
| ITGB3BP | 0.622643645 | 0.879442691 |
| KARS | 0.608261498 | 0.91236092 |
| KATNA1 | 0.60604637 | 0.86906548 |
| KDM1A | 0.616784119 | 0.883100158 |
| KHDRBS1 | 0.656432039 | 0.870278366 |
| KIAA0020 | 0.602980715 | 0.835530859 |
| KPNB1 | 0.603374353 | 0.859585963 |
| L3MBTL2 | 0.602721469 | 0.863537264 |
| LAS1L | 0.655624119 | 0.923307962 |
| LMNB1 | 0.617224811 | 0.811128987 |
| LSM5 | 0.671334266 | 0.897307672 |
| LTV1 | 0.639710246 | 0.861448797 |
| MAGOH | 0.686400577 | 0.855294959 |
| MAGOHB | 0.662073496 | 0.877616596 |
| MCM2 | 0.634036215 | 0.863052185 |
| MCM3 | 0.690881756 | 0.915466348 |
| MCM4 | 0.662219496 | 0.809132651 |
| MCM6 | 0.617305739 | 0.822127024 |
| MCM7 | 0.671701046 | 0.883990954 |
| METAP2 | 0.61241123 | 0.85421137 |
| METTL13 | 0.635474023 | 0.885140202 |
| MIOS | 0.606867131 | 0.813326581 |
| MIS18A | 0.623559903 | 0.86677086 |
| MKI67IP | 0.645773165 | 0.884854809 |
| MLH1 | 0.624727258 | 0.908350079 |
| MNAT1 | 0.608292809 | 0.855612734 |
| MRPL50 | 0.648265808 | 0.902302416 |
| MRPS23 | 0.616311394 | 0.942206508 |
| MRPS27 | 0.641803298 | 0.92653299 |
| MRTO4 | 0.613355014 | 0.918573667 |
| MSH6 | 0.602907147 | 0.810138606 |
| MTA2 | 0.649391195 | 0.924495229 |
| MTERFD1 | 0.648457739 | 0.915560331 |
| MTHFD1 | 0.629250188 | 0.849784989 |
| MZT1 | 0.602970843 | 0.816634613 |
| NAA38 | 0.654217025 | 0.847150319 |
| NAP1L4 | 0.615965454 | 0.888227912 |
| NASP | 0.684138406 | 0.9078895 |
| NAT10 | 0.621643756 | 0.871343776 |
| NCAPD3 | 0.620548373 | 0.817376972 |
| NCL | 0.706022973 | 0.903381027 |
| NFYB | 0.612569627 | 0.804501277 |
| NGDN | 0.600880486 | 0.84142805 |
| NHP2L1 | 0.624325738 | 0.907874505 |
| NIF3L1 | 0.647334455 | 0.875279166 |
| NIT2 | 0.611929977 | 0.897470401 |
| NOL11 | 0.616035806 | 0.801494053 |
| NOLC1 | 0.631138689 | 0.860023306 |
| NOP14 | 0.601954837 | 0.89351581 |
| NOP56 | 0.605910191 | 0.872933406 |
| NOP58 | 0.647841851 | 0.89565414 |
| NOP9 | 0.6401865 | 0.858415991 |
| NPM1 | 0.626010712 | 0.909476045 |
| NPM3 | 0.601410366 | 0.860822939 |
| NR2C2AP | 0.604596099 | 0.854093673 |
| NSMCE2 | 0.607971656 | 0.891326643 |
| NSUN2 | 0.613897677 | 0.845029316 |
| NUDT5 | 0.698798386 | 0.926445487 |
| NUP107 | 0.665671361 | 0.867939088 |
| NUP205 | 0.623716885 | 0.824615834 |
| NUP35 | 0.664532293 | 0.871277707 |
| NUP37 | 0.654107233 | 0.933208408 |
| NUP85 | 0.667544005 | 0.927569276 |
| NUP88 | 0.657263295 | 0.901438529 |
| NUP93 | 0.671467103 | 0.935965158 |
| PA2G4 | 0.665630349 | 0.891664879 |
| PAICS | 0.612608944 | 0.820577932 |
| PAK1IP1 | 0.605927802 | 0.848880519 |
| PARK7 | 0.606202621 | 0.916963516 |
| PCNA | 0.693443393 | 0.911612616 |
| PDCD2 | 0.623753823 | 0.872490664 |
| PES1 | 0.610552946 | 0.925374544 |
| PFDN6 | 0.625122048 | 0.860447393 |
| PGAM5 | 0.618312342 | 0.810168982 |
| PHF5A | 0.655525163 | 0.864121363 |
| POLA2 | 0.616312633 | 0.817399393 |
| POLE2 | 0.664836168 | 0.811024416 |
| POLE3 | 0.659877009 | 0.946845524 |
| POLR1C | 0.607857996 | 0.904281848 |
| POLR2K | 0.659082596 | 0.861049941 |
| POP1 | 0.621224461 | 0.83160105 |
| PPIA | 0.620360426 | 0.895836342 |
| PPIH | 0.666554753 | 0.908104784 |
| PPM1G | 0.614638686 | 0.917256351 |
| PRIM1 | 0.603075202 | 0.819827543 |
| PRIM2 | 0.652440698 | 0.871402738 |
| PRMT1 | 0.609970293 | 0.903309662 |
| PRMT3 | 0.606907281 | 0.818004181 |
| PRPF19 | 0.684947563 | 0.894579614 |
| PRPF4 | 0.618175135 | 0.895145715 |
| PSMA1 | 0.642535928 | 0.872760726 |
| PSMC1 | 0.607162673 | 0.849996032 |
| PSMD13 | 0.607398313 | 0.898016697 |
| PSMD14 | 0.606139454 | 0.863180032 |
| PSMD6 | 0.613168732 | 0.92177601 |
| PSPC1 | 0.648761835 | 0.853065037 |
| PTBP1 | 0.636197789 | 0.865553565 |
| PTMA | 0.67101775 | 0.905055133 |
| PUS1 | 0.607307155 | 0.860639739 |
| RAB28 | 0.629419974 | 0.817870668 |
| RAD51 | 0.618211639 | 0.816434856 |
| RAE1 | 0.610417495 | 0.901557121 |
| RAN | 0.655227665 | 0.950661581 |
| RANBP1 | 0.633452228 | 0.904388145 |
| RBBP7 | 0.663734487 | 0.929503294 |
| RBM17 | 0.642792066 | 0.845654338 |
| RBM8A | 0.613177444 | 0.819332644 |
| RCC1 | 0.636599001 | 0.877320948 |
| RCC2 | 0.633609668 | 0.872703776 |
| RFC4 | 0.660782964 | 0.879927499 |
| RFC5 | 0.693088643 | 0.862785855 |
| RFT1 | 0.624149785 | 0.889271009 |
| RFWD3 | 0.618847973 | 0.83993384 |
| RHNO1 | 0.603729083 | 0.866459321 |
| RIOK1 | 0.610552736 | 0.853679775 |
| RMI2 | 0.604043022 | 0.823244185 |
| RNPS1 | 0.628633261 | 0.898960268 |
| RPF1 | 0.607500145 | 0.868486837 |
| RPP30 | 0.662451922 | 0.914973262 |
| RQCD1 | 0.637127055 | 0.893966127 |
| RRM1 | 0.611343999 | 0.829010572 |
| RRP15 | 0.631564445 | 0.836103136 |
| RUVBL1 | 0.602569349 | 0.919134381 |
| SAAL1 | 0.677353531 | 0.925956816 |
| SAE1 | 0.625683407 | 0.936293362 |
| SCFD2 | 0.634161606 | 0.810667631 |
| SERBP1 | 0.605139963 | 0.815356843 |
| SET | 0.68981737 | 0.882321797 |
| SF3A3 | 0.650631341 | 0.92190142 |
| SF3B3 | 0.618611683 | 0.83723961 |
| SF3B4 | 0.602789287 | 0.828410127 |
| SFPQ | 0.663142486 | 0.825366315 |
| SKA2 | 0.620121484 | 0.8525576 |
| SLBP | 0.691893724 | 0.890203205 |
| SMARCD2 | 0.605248284 | 0.831958849 |
| SMPD4 | 0.631353837 | 0.916011342 |
| SNRNP40 | 0.647969036 | 0.949108063 |
| SNRPA | 0.609886584 | 0.849665416 |
| SNRPB2 | 0.675116116 | 0.926422589 |
| SNRPD1 | 0.682735473 | 0.941098422 |
| SNRPD3 | 0.670152907 | 0.846636847 |
| SNRPE | 0.671224498 | 0.8964033 |
| SNRPF | 0.655175857 | 0.876520383 |
| SNRPG | 0.635736508 | 0.870013442 |
| SPDL1 | 0.616561623 | 0.834619221 |
| SRRT | 0.652394467 | 0.890719253 |
| SRSF1 | 0.722358104 | 0.921792503 |
| SRSF2 | 0.700490305 | 0.848224859 |
| SRSF3 | 0.700316004 | 0.912035402 |
| SRSF7 | 0.689701763 | 0.85446275 |
| SRSF9 | 0.614122713 | 0.870442147 |
| SSB | 0.640130219 | 0.895195104 |
| SSBP1 | 0.627941221 | 0.923833805 |
| SSRP1 | 0.658675839 | 0.950004781 |
| SUMO2 | 0.635626227 | 0.859290511 |
| SUPT16H | 0.639433168 | 0.846510326 |
| SUV39H1 | 0.612600915 | 0.876784621 |
| TAF9 | 0.641118813 | 0.881978637 |
| TARDBP | 0.677023099 | 0.887046118 |
| TBCA | 0.602681668 | 0.857034975 |
| TCF19 | 0.600108196 | 0.86456533 |
| TCOF1 | 0.636892368 | 0.89913274 |
| TFDP1 | 0.651171524 | 0.878284805 |
| TIMELESS | 0.628266834 | 0.845278485 |
| TIMM21 | 0.610805032 | 0.820594098 |
| TIPIN | 0.638079393 | 0.853519082 |
| TRA2B | 0.717275684 | 0.885882576 |
| TRAIP | 0.610305879 | 0.849483892 |
| TRMT10C | 0.605689717 | 0.838226769 |
| TRMT6 | 0.61259335 | 0.826501747 |
| TSEN15 | 0.613674618 | 0.830190805 |
| TSN | 0.610274986 | 0.839394613 |
| TSR1 | 0.60428225 | 0.872919693 |
| TTC27 | 0.618929525 | 0.861991074 |
| TTI2 | 0.613331366 | 0.894451329 |
| TUBA1B | 0.62556813 | 0.854382252 |
| TUBB | 0.620343535 | 0.873204809 |
| U2AF1 | 0.673901212 | 0.878793126 |
| U2AF2 | 0.652124997 | 0.863392569 |
| UBAP2L | 0.606782543 | 0.840709481 |
| UBE2N | 0.661967565 | 0.872592034 |
| UBE2T | 0.604518954 | 0.843289579 |
| UMPS | 0.640517617 | 0.880662486 |
| USP39 | 0.654298161 | 0.927457081 |
| USP5 | 0.645761487 | 0.90751919 |
| UTP18 | 0.604175898 | 0.882291583 |
| UTP3 | 0.602439854 | 0.824207234 |
| UTP6 | 0.633139101 | 0.886712913 |
| VBP1 | 0.634874736 | 0.859312727 |
| VRK1 | 0.663031958 | 0.905020705 |
| WBP11 | 0.609705241 | 0.843284465 |
| WBSCR16 | 0.602008263 | 0.87322689 |
| WBSCR22 | 0.631884239 | 0.904052807 |
| WDR61 | 0.679057297 | 0.893947552 |
| WDR75 | 0.602088495 | 0.855403308 |
| XRCC3 | 0.614490103 | 0.828081741 |
| XRCC5 | 0.624164642 | 0.861647246 |
| XRCC6 | 0.67195287 | 0.925927442 |
| YARS2 | 0.61705931 | 0.907650448 |
| YBX1 | 0.613744096 | 0.85948618 |
| YEATS4 | 0.602390292 | 0.808240645 |
| ZW10 | 0.62855117 | 0.850541064 |
| ZWINT | 0.611938279 | 0.808112673 |
