# Supplementary material for: m6A Modification Mediates Mucosal Immune Microenvironment and Therapeutic Response in Inflammatory Bowel Disease
Source: Front Cell Dev Biol. 2021 Aug 6;9:692160. doi: 10.3389/fcell.2021.692160 (PMC8378837; doi:10.3389/fcell.2021.692160)
Supplement: Supplementary file 7 [file Table_6.DOC]

Supplementary Table 6. The key gene information obtained from the hub genes of turquoise module overlapping with the PPI

| hub_gene | Gene significance | | Module membership | PPI_degree |
| --- | --- | --- | --- | --- |
| ALYREF | 0.610778225 | 0.882706583 | | 234 |
| CDC23 | 0.60809821 | 0.836474811 | | 284 |
| CDK2 | 0.608776621 | 0.830625059 | | 225 |
| CPSF3 | 0.628416554 | 0.93650412 | | 221 |
| H2AFZ | 0.655212736 | 0.894871067 | | 289 |
| HNRNPC | 0.684325131 | 0.895056917 | | 220 |
| MAGOH | 0.686400577 | 0.855294959 | | 299 |
| MAGOHB | 0.662073496 | 0.877616596 | | 297 |
| NHP2L1 | 0.624325738 | 0.907874505 | | 321 |
| NUP107 | 0.665671361 | 0.867939088 | | 216 |
| NUP37 | 0.654107233 | 0.933208408 | | 214 |
| POLR2K | 0.659082596 | 0.861049941 | | 314 |
| PRPF19 | 0.684947563 | 0.894579614 | | 235 |
| RBBP7 | 0.663734487 | 0.929503294 | | 261 |
| RBM8A | 0.613177444 | 0.819332644 | | 302 |
| RNPS1 | 0.628633261 | 0.898960268 | | 325 |
| SNRPD1 | 0.682735473 | 0.941098422 | | 234 |
| SNRPD3 | 0.670152907 | 0.846636847 | | 241 |
| SNRPE | 0.671224498 | 0.8964033 | | 247 |
| SNRPF | 0.655175857 | 0.876520383 | | 238 |
| SNRPG | 0.635736508 | 0.870013442 | | 241 |
| SRRT | 0.652394467 | 0.890719253 | | 219 |
| SRSF1 | 0.722358104 | 0.921792503 | | 236 |
| SRSF2 | 0.700490305 | 0.848224859 | | 226 |
| SRSF3 | 0.700316004 | 0.912035402 | | 228 |
| SRSF7 | 0.689701763 | 0.85446275 | | 226 |
| SRSF9 | 0.614122713 | 0.870442147 | | 218 |
| U2AF2 | 0.652124997 | 0.863392569 | | 232 |
| UBE2N | 0.661967565 | 0.872592034 | | 352 |
