# Supplementary material for: m6A Modification Mediates Mucosal Immune Microenvironment and Therapeutic Response in Inflammatory Bowel Disease
Source: Front Cell Dev Biol. 2021 Aug 6;9:692160. doi: 10.3389/fcell.2021.692160 (PMC8378837; doi:10.3389/fcell.2021.692160)
Supplement: Supplementary file 8 [file Table_7.DOC]

Supplementary Table 7. Consistency cluster analysis for IBD samples

| Sample | m6Acluster | Source | Biopsy location | Disease status | Gender |
| --- | --- | --- | --- | --- | --- |
| GSM3043377 | m6Acluster1 | colon | Sigmoid Colon | CD | Female |
| GSM3043378 | m6Acluster1 | intestinal | Ileum | CD | Female |
| GSM3043379 | m6Acluster1 | rectum | Rectum | CD | Female |
| GSM3043380 | m6Acluster2 | colon | Sigmoid Colon | CD | Female |
| GSM3043381 | m6Acluster1 | intestinal | Ileum | UC | Female |
| GSM3043382 | m6Acluster2 | rectum | Rectum | UC | Female |
| GSM3043383 | m6Acluster2 | colon | Sigmoid Colon | UC | Female |
| GSM3043384 | m6Acluster2 | colon | Sigmoid Colon | UC | Female |
| GSM3043385 | m6Acluster1 | intestinal | Ileum | UC | Female |
| GSM3043386 | m6Acluster1 | rectum | Rectum | UC | Female |
| GSM3043387 | m6Acluster1 | intestinal | Ileum | UC | Female |
| GSM3043388 | m6Acluster2 | rectum | Rectum | UC | Female |
| GSM3043389 | m6Acluster2 | colon | Sigmoid Colon | UC | Female |
| GSM3043390 | m6Acluster1 | colon | Sigmoid Colon | UC | Male |
| GSM3043391 | m6Acluster2 | intestinal | Ileum | UC | Male |
| GSM3043392 | m6Acluster1 | rectum | Rectum | UC | Male |
| GSM3043393 | m6Acluster2 | colon | Sigmoid Colon | UC | Male |
| GSM3043394 | m6Acluster1 | intestinal | Ileum | CD | Female |
| GSM3043395 | m6Acluster2 | rectum | Rectum | CD | Female |
| GSM3043396 | m6Acluster1 | colon | Sigmoid Colon | CD | Female |
| GSM3043397 | m6Acluster1 | intestinal | Ileum | CD | Female |
| GSM3043398 | m6Acluster2 | rectum | Rectum | CD | Female |
| GSM3043399 | m6Acluster1 | colon | Sigmoid Colon | CD | Female |
| GSM3043400 | m6Acluster1 | intestinal | Ileum | UC | Female |
| GSM3043401 | m6Acluster1 | rectum | Rectum | UC | Female |
| GSM3043402 | m6Acluster1 | colon | Sigmoid Colon | UC | Female |
| GSM3043403 | m6Acluster2 | intestinal | Ileum | CD | Female |
| GSM3043404 | m6Acluster1 | rectum | Rectum | CD | Female |
| GSM3043405 | m6Acluster2 | colon | Sigmoid Colon | CD | Female |
| GSM3043406 | m6Acluster1 | colon | Sigmoid Colon | UC | Female |
| GSM3043407 | m6Acluster2 | intestinal | Ileum | UC | Female |
| GSM3043408 | m6Acluster2 | rectum | Rectum | UC | Female |
| GSM3043409 | m6Acluster1 | intestinal | Cecum | UC | Female |
| GSM3043410 | m6Acluster1 | intestinal | Ileum | UC | Female |
| GSM3043411 | m6Acluster2 | rectum | Rectum | UC | Female |
| GSM3043412 | m6Acluster1 | intestinal | Ileum | CD | Female |
| GSM3043413 | m6Acluster1 | rectum | Rectum | CD | Female |
| GSM3043414 | m6Acluster1 | colon | Sigmoid Colon | CD | Female |
| GSM3043415 | m6Acluster1 | intestinal | Ileum | CD | Male |
| GSM3043416 | m6Acluster2 | rectum | Rectum | CD | Male |
| GSM3043417 | m6Acluster2 | colon | Sigmoid Colon | CD | Male |
| GSM3043418 | m6Acluster1 | colon | Descending (left-sided) colon | CD | Female |
| GSM3043419 | m6Acluster1 | intestinal | Ileum | CD | Female |
| GSM3043420 | m6Acluster2 | rectum | Rectum | CD | Female |
| GSM3043421 | m6Acluster2 | intestinal | Cecum | CD | Female |
| GSM3043424 | m6Acluster2 | intestinal | Terminal ileum | CD | Male |
| GSM3043425 | m6Acluster1 | rectum | Ileum | CD | Male |
| GSM3043426 | m6Acluster2 | rectum | Rectum | CD | Male |
| GSM3043427 | m6Acluster1 | intestinal | Ileum | CD | Female |
| GSM3043428 | m6Acluster2 | rectum | Rectum | CD | Female |
| GSM3043429 | m6Acluster1 | colon | Sigmoid Colon | CD | Female |
| GSM3043431 | m6Acluster2 | rectum | Rectum | CD | Male |
| GSM3043432 | m6Acluster2 | colon | Descending (left-sided) colon | UC | Male |
| GSM3043433 | m6Acluster1 | intestinal | Ileum | UC | Male |
| GSM3043434 | m6Acluster1 | rectum | Rectum | UC | Male |
| GSM3043435 | m6Acluster2 | colon | Transverse colon | UC | Male |
| GSM3043436 | m6Acluster1 | intestinal | Terminal ileum | CD | Male |
| GSM3043437 | m6Acluster1 | intestinal | Ileum | CD | Male |
| GSM3043438 | m6Acluster2 | rectum | Rectum | CD | Male |
| GSM3043440 | m6Acluster1 | intestinal | Ileum | CD | Female |
| GSM3043441 | m6Acluster2 | rectum | Rectum | CD | Female |
| GSM3043442 | m6Acluster2 | colon | Sigmoid Colon | CD | Female |
| GSM3043443 | m6Acluster1 | intestinal | Ileum | UC | Female |
| GSM3043444 | m6Acluster2 | rectum | Rectum | UC | Female |
| GSM3043445 | m6Acluster1 | colon | Sigmoid Colon | UC | Female |
| GSM3043446 | m6Acluster1 | intestinal | Ileum | UC | Female |
| GSM3043447 | m6Acluster1 | rectum | Rectum | UC | Female |
| GSM3043448 | m6Acluster2 | colon | Ascending (right-sided) colon | UC | Female |
| GSM3043449 | m6Acluster2 | intestinal | Terminal ileum | CD | Male |
| GSM3043450 | m6Acluster1 | intestinal | Ileum | CD | Male |
| GSM3043451 | m6Acluster1 | rectum | Rectum | CD | Male |
| GSM3043452 | m6Acluster2 | colon | Sigmoid Colon | UC | Female |
| GSM3043453 | m6Acluster1 | intestinal | Ileum | UC | Female |
| GSM3043454 | m6Acluster2 | rectum | Rectum | UC | Female |
| GSM3043455 | m6Acluster2 | colon | Transverse colon | UC | Female |
| GSM3043456 | m6Acluster2 | intestinal | Ileum | UC | Female |
| GSM3043457 | m6Acluster1 | rectum | Rectum | UC | Female |
| GSM3043458 | m6Acluster2 | intestinal | Cecum | CD | Female |
| GSM3043459 | m6Acluster2 | intestinal | Ileum | CD | Female |
| GSM3043460 | m6Acluster1 | rectum | Rectum | CD | Female |
| GSM3043461 | m6Acluster2 | colon | Descending (left-sided) colon | CD | Female |
| GSM3043462 | m6Acluster1 | intestinal | Ileum | CD | Female |
| GSM3043463 | m6Acluster1 | rectum | Rectum | CD | Female |
| GSM3043464 | m6Acluster2 | colon | Sigmoid Colon | CD | Male |
| GSM3043465 | m6Acluster1 | rectum | Rectum | CD | Male |
| GSM3043466 | m6Acluster2 | colon | Ascending (right-sided) colon | CD | Male |
| GSM3043467 | m6Acluster1 | rectum | Rectum | CD | Male |
| GSM3043468 | m6Acluster1 | intestinal | Ileum | CD | Male |
| GSM3043469 | m6Acluster2 | rectum | Rectum | CD | Male |
| GSM3043470 | m6Acluster1 | colon | Ascending (right-sided) colon | CD | Male |
| GSM3043471 | m6Acluster2 | intestinal | Ileum | CD | Male |
| GSM3043472 | m6Acluster2 | rectum | Rectum | CD | Male |
| GSM3043473 | m6Acluster2 | intestinal | Ileum | CD | Female |
| GSM3043474 | m6Acluster2 | rectum | Rectum | CD | Female |
| GSM3043475 | m6Acluster1 | intestinal | Ileum | CD | Female |
| GSM3043476 | m6Acluster2 | rectum | Rectum | CD | Female |
| GSM3043483 | m6Acluster1 | intestinal | Ileum | UC | Male |
| GSM3043484 | m6Acluster1 | rectum | Rectum | UC | Male |
| GSM3043487 | m6Acluster2 | intestinal | Ileum | CD | Female |
| GSM3043488 | m6Acluster2 | rectum | Rectum | CD | Female |
| GSM3043489 | m6Acluster2 | colon | Descending (left-sided) colon | CD | Male |
| GSM3043490 | m6Acluster1 | intestinal | Ileum | CD | Male |
| GSM3043491 | m6Acluster1 | rectum | Rectum | CD | Male |
| GSM3043495 | m6Acluster2 | colon | Ascending (right-sided) colon | CD | Female |
| GSM3043496 | m6Acluster2 | intestinal | Ileum | CD | Female |
| GSM3043497 | m6Acluster2 | rectum | Rectum | CD | Female |
| GSM3043501 | m6Acluster2 | intestinal | Ileum | UC | Female |
| GSM3043502 | m6Acluster2 | rectum | Rectum | UC | Female |
| GSM3043503 | m6Acluster1 | colon | Transverse colon | UC | Female |
| GSM3043504 | m6Acluster1 | intestinal | Ileum | UC | Female |
| GSM3043505 | m6Acluster2 | rectum | Rectum | UC | Female |
| GSM3043506 | m6Acluster2 | colon | Transverse colon | UC | Female |
| GSM3043507 | m6Acluster2 | intestinal | Ileum | CD | Male |
| GSM3043508 | m6Acluster1 | rectum | Rectum | CD | Male |
| GSM3043518 | m6Acluster2 | colon | Descending (left-sided) colon | UC | Male |
| GSM3043519 | m6Acluster2 | intestinal | Ileum | UC | Male |
| GSM3043520 | m6Acluster2 | rectum | Rectum | UC | Male |
| GSM3043521 | m6Acluster1 | intestinal | Ileum | CD | Female |
| GSM3043522 | m6Acluster1 | rectum | Rectum | CD | Female |
| GSM3043523 | m6Acluster1 | colon | Transverse colon | CD | Female |
| GSM3043524 | m6Acluster1 | intestinal | Ileum | CD | Male |
| GSM3043525 | m6Acluster1 | rectum | Rectum | CD | Male |
| GSM3043526 | m6Acluster2 | intestinal | Ileum | CD | Male |
| GSM3043527 | m6Acluster1 | rectum | Rectum | CD | Male |
| GSM3043528 | m6Acluster1 | colon | Transverse colon | CD | Male |
| GSM3043529 | m6Acluster2 | colon | Sigmoid Colon | CD | Male |
| GSM3043530 | m6Acluster2 | intestinal | Ileum | CD | Male |
| GSM3043531 | m6Acluster1 | rectum | Rectum | CD | Male |
| GSM3043532 | m6Acluster2 | colon | Descending (left-sided) colon | UC | Male |
| GSM3043533 | m6Acluster1 | intestinal | Ileum | UC | Male |
| GSM3043534 | m6Acluster1 | rectum | Rectum | UC | Male |
| GSM3043535 | m6Acluster2 | rectum | Cecum | CD | Male |
| GSM3043536 | m6Acluster2 | intestinal | Ileum | CD | Male |
| GSM3043537 | m6Acluster2 | rectum | Rectum | CD | Male |
| GSM3043538 | m6Acluster2 | colon | Ascending (right-sided) colon | CD | Female |
| GSM3043539 | m6Acluster2 | intestinal | Ileum | CD | Female |
| GSM3043540 | m6Acluster1 | rectum | Rectum | CD | Female |
| GSM3043541 | m6Acluster1 | intestinal | Ileum | UC | Female |
| GSM3043542 | m6Acluster1 | rectum | Rectum | UC | Female |
| GSM3043544 | m6Acluster2 | rectum | Rectum | UC | Male |
| GSM3043545 | m6Acluster2 | colon | Descending (left-sided) colon | UC | Male |
| GSM3043546 | m6Acluster1 | colon | Transverse colon | CD | Female |
| GSM3043547 | m6Acluster2 | intestinal | Ileum | CD | Female |
| GSM3043548 | m6Acluster1 | rectum | Rectum | CD | Female |
| GSM3043549 | m6Acluster2 | intestinal | Ileum | UC | Male |
| GSM3043550 | m6Acluster2 | rectum | Rectum | UC | Male |
| GSM3043554 | m6Acluster1 | intestinal | Ileum | CD | Female |
| GSM3043555 | m6Acluster1 | rectum | Rectum | CD | Female |
| GSM3043556 | m6Acluster1 | colon | Transverse colon | CD | Female |
| GSM3043557 | m6Acluster1 | rectum | Rectum | CD | Female |
| GSM3043558 | m6Acluster1 | rectum | Rectum | CD | Female |
| GSM3043559 | m6Acluster2 | colon | Descending (left-sided) colon | CD | Female |
| GSM3043560 | m6Acluster1 | intestinal | Ileum | CD | Male |
| GSM3043561 | m6Acluster2 | rectum | Rectum | CD | Male |
| GSM3043562 | m6Acluster2 | rectum | Rectum | CD | Male |
| GSM3043563 | m6Acluster1 | rectum | Rectum | CD | Male |
| GSM3043564 | m6Acluster1 | rectum | Cecum | CD | Male |
| GSM3043565 | m6Acluster1 | intestinal | Ileum | CD | Male |
| GSM3043566 | m6Acluster1 | intestinal | Ileum | CD | Female |
| GSM3043567 | m6Acluster1 | rectum | Rectum | CD | Female |
| GSM3043568 | m6Acluster2 | colon | Sigmoid Colon | CD | Female |
| GSM3043569 | m6Acluster2 | intestinal | Ileum | UC | Female |
| GSM3043570 | m6Acluster1 | rectum | Rectum | UC | Female |
| GSM3043571 | m6Acluster2 | colon | Ascending (right-sided) colon | UC | Female |
| GSM3043572 | m6Acluster1 | intestinal | Ileum | CD | Male |
| GSM3043573 | m6Acluster1 | rectum | Rectum | CD | Male |
| GSM3043574 | m6Acluster2 | intestinal | Ileum | CD | Female |
| GSM3043575 | m6Acluster2 | rectum | Rectum | CD | Female |
| GSM3043576 | m6Acluster1 | intestinal | Ileum | CD | Female |
| GSM3043577 | m6Acluster1 | rectum | Rectum | CD | Female |
| GSM3043578 | m6Acluster2 | intestinal | Ileum | CD | Female |
| GSM3043579 | m6Acluster1 | rectum | Rectum | CD | Female |
| GSM3043592 | m6Acluster2 | colon | Transverse colon | UC | Male |
| GSM3043593 | m6Acluster1 | colon | Ascending (right-sided) colon | UC | Male |
| GSM3043594 | m6Acluster1 | rectum | Rectum | UC | Male |
| GSM3043595 | m6Acluster1 | intestinal | Ileum | CD | Female |
| GSM3043596 | m6Acluster2 | rectum | Rectum | CD | Female |
| GSM3043597 | m6Acluster2 | intestinal | Ileum | UC | Female |
| GSM3043598 | m6Acluster1 | rectum | Rectum | UC | Female |
| GSM3043604 | m6Acluster1 | intestinal | Ileum | CD | Male |
| GSM3043605 | m6Acluster1 | rectum | Rectum | CD | Male |
| GSM3043606 | m6Acluster1 | intestinal | Ileum | CD | Male |
| GSM3043607 | m6Acluster2 | rectum | Rectum | CD | Male |
| GSM3043608 | m6Acluster1 | intestinal | Ileum | UC | Female |
| GSM3043609 | m6Acluster2 | rectum | Rectum | UC | Female |
| GSM3043610 | m6Acluster2 | intestinal | Ileum | UC | Male |
| GSM3043611 | m6Acluster2 | rectum | Rectum | UC | Male |
| GSM3043614 | m6Acluster2 | colon | Transverse colon | CD | Male |
| GSM3043615 | m6Acluster1 | rectum | Rectum | CD | Male |
| GSM3043616 | m6Acluster2 | intestinal | Ileum | CD | Male |
| GSM3043617 | m6Acluster2 | rectum | Rectum | CD | Male |
| GSM3043622 | m6Acluster2 | intestinal | Ileum | CD | Male |
| GSM3043623 | m6Acluster2 | rectum | Rectum | CD | Male |
| GSM3043624 | m6Acluster2 | intestinal | Ileum | UC | Male |
| GSM3043625 | m6Acluster2 | rectum | Rectum | UC | Male |
| GSM3043626 | m6Acluster2 | intestinal | Ileum | CD | Male |
| GSM3043627 | m6Acluster1 | rectum | Rectum | CD | Male |
